# Neuronal Death in the CNS Autonomic Control Center Comes Very Early after Cardiac Arrest and Is Not Significantly Attenuated by Prompt Hypothermic Treatment in Rats

**DOI:** 10.3390/cells10010060

**Published:** 2021-01-02

**Authors:** Ji Hyeon Ahn, Tae-Kyeong Lee, Hyun-Jin Tae, Bora Kim, Hyejin Sim, Jae-Chul Lee, Dae Won Kim, Yoon Sung Kim, Myoung Cheol Shin, Yoonsoo Park, Jun Hwi Cho, Joon Ha Park, Choong-Hyun Lee, Soo Young Choi, Moo-Ho Won

**Affiliations:** 1 Department of Physical Therapy, College of Health Science, Youngsan University, Yangsan 50510, Korea; jh-ahn@ysu.ac.kr; 2 Department of Neurobiology, School of Medicine, Kangwon National University, Chuncheon 24341, Korea; nbrkim17@gmail.com (B.K.); janny20@naver.com (H.S.); anajclee@kangwon.ac.kr (J.-C.L.); 3 Department of Biomedical Science and Research Institute for Bioscience and Biotechnology, Hallym University, Chuncheon 24252, Korea; tk-lee@hallym.ac.kr; 4 Bio-Safety Research Institute, College of Veterinary Medicine, Chonbuk National University, Iksan 54596, Korea; anatotae@gmail.com; 5 Department of Biochemistry and Molecular Biology, and Research Institute of Oral Sciences, College of Dentistry, Gangnung-Wonju National University, Gangneung 25457, Korea; kimdw@gwnu.ac.kr; 6 Department of Emergency Medicine, Samcheok Medical Center, Samcheok 25920, Korea; kys99008@Kangwon.ac.kr; 7 Department of Emergency Medicine, School of Medicine, Kangwon National University, Chuncheon 24341, Korea; dr10126@naver.com (M.C.S.); pyoonsoo@naver.com (Y.P.); cjhemd@kangwon.ac.kr (J.H.C.); 8 Department of Anatomy, College of Korean Medicine, Dongguk University, Gyeongju 38066, Korea; jh-park@dongguk.ac.kr; 9 Department of Pharmacy, College of Pharmacy, Dankook University, Cheonan 31116, Korea; anaphy@dankook.ac.kr

**Keywords:** cardiopulmonary resuscitation, Fluoro-Jade B, prompt hypothermia, neuronal death, autonomic lower motor neurons, myelencephalon, spinal cord

## Abstract

Autonomic dysfunction in the central nervous system (CNS) can cause death after recovery from a cardiac arrest (CA). However, few studies on histopathological changes in animal models of CA have been reported. In this study, we investigated the prevalence of neuronal death and damage in various brain regions and the spinal cord at early times after asphyxial CA and we studied the relationship between the mortality rate and neuronal damage following hypothermic treatment after CA. Rats were subjected to 7–8 min of asphyxial CA, followed by resuscitation and prompt hypothermic treatment. Eight regions related to autonomic control (the cingulate cortex, hippocampus, thalamus, hypothalamus, myelencephalon, and spinal cord) were examined using cresyl violet (a marker for Nissl substance) and Fluoro-Jade B (a marker for neuronal death). The survival rate was 44.5% 1 day post-CA, 18.2% 2 days post-CA and 0% 5 days post-CA. Neuronal death started 12 h post-CA in the gigantocellular reticular nucleus and caudoventrolateral reticular nucleus in the myelencephalon and lamina VII in the cervical, thoracic, lumbar, and sacral spinal cord, of which neurons are related to autonomic lower motor neurons. In these regions, Iba-1 immunoreactivity indicating microglial activation (microgliosis) was gradually increased with time after CA. Prompt hypothermic treatment increased the survival rate at 5 days after CA with an attenuation of neuronal damages and death in the damaged regions. However, the survival rate was 0% at 12 days after CA. Taken together, our study suggests that the early damage and death of neurons related to autonomic lower motor neurons was significantly related to the high mortality rate after CA and that prompt hypothermic therapy could increase the survival rate temporarily after CA, but could not ultimately save the animal.

## 1. Introduction

Cardiac arrest (CA) is mainly caused by lack of oxygen (asphyxia), and ventricular fibrillation and is one of the major causes of death [1]. A large number of patients with CA do not survive long or experience neurological disabilities due to ischemia-reperfusion (IR) injury [2,3]. In particular, airway obstruction, respiratory failure, pulmonary embolism, drowning, and choking are common causes of asphyxial CA [4]. A rat model of asphyxial CA was developed for brain resuscitation studies [5,6,7]. However, the mortality rate within one day after the return of spontaneous circulation (RoSC) after asphyxial CA was very high, more than 50% [5].

It has been suggested that postcardiac arrest syndrome, which includes brain damage (post-resuscitation encephalopathy) and extracranial multiple organ dysfunction during the post-resuscitation phase after CA, is highly correlated with low survival rate [1,8]. In addition, Li et al. (2015) reported that the immediate activation of cortico-cardiac signaling could deteriorate cardiac arrhythmia during asphyxia and cause the death of rats after asphyxial CA [4]. Recently, cellular changes associated with IR injury (such as an increase of inflammatory cytokines and the opening of mitochondrial permeability transition pores) in CA were suggested to be a modifiable therapeutic target for neuroprotection in patients undergoing cardiopulmonary resuscitation, and hypothermic therapy or remote ischemic conditioning could reduce the potential harm of IR injury [3].

To find potential therapeutic targets for neuroprotection and increase the survival rate after CA, it is important to investigate when and where neuronal damage and death occurs in the central nervous system (brain and spinal cord). Despite the important role of the myelencephalon in survival and death after brain insults [9], there has been less evidence for the role of neuronal damage and death in the central nervous system (CNS) autonomic control center. Most of the previous studies on ischemic brain damage after asphyxial CA have been conducted in confined areas such as the cerebral cortex, hippocampus and cerebellum using the TUNEL method [2] or morphological analyses using hematoxylin and eosin staining [1,10] and toluidine blue staining [11].

Therefore, we examined the prevalence of neuronal damage/death in important brain regions (including in the cortex, striatum, thalamus, hypothalamus, hippocampus, substantia nigra, and myelencephalon) and spinal cord using Fluoro-Jade B (F-JB), which is an excellent marker for neuronal degeneration and death [12,13]. In addition, we investigated the relationship between neuronal damage and death and the mortality rate of rats after asphyxial CA, and estimated survival rate from applying prompt hypothermic therapy after CA.

## 2. Materials and Methods

### 2.1. Animals

We used 108 male rats (Sprague-Dawley; body weight, 300–350 g) at 16 weeks of age, which were purchased from Central Lab Animal Inc. (Seoul, Republic of Korea). Animal handling and care adhered to the Guide for the Care and Use of Laboratory Animals (The National CA demies Press, 8th ed., 2011). The protocol of this experiment was approved by the Kangwon National University-Institutional Animal Care and Use Committee (approval no. KW-200113-1). Rats were randomly assigned to three groups as follows: (1) sham operated group (sham group, *n* = 5), which received no asphyxial CA operation, (2) CA operated group (CA group, *n* = 9 at each point in time, *n* = 11 at 2 days), which received asphyxial CA operation and the control of normal body temperature (37 ± 0.5 °C) for 4 h after RoSC and was sacrificed at 12 h, 1 day and 2 days after RoSC, (3) CA operated and hypothermia treated group (CA/hypothermia group, *n* = 9 at each point in time), which received CA operation and hypothermic therapy (33.0 ± 0.5 °C) for 4 h and was sacrificed at 12 h, 1 day and 2 days after RoSC. In order to check the survival rate after RoSC, 9 animals were used at each point in time after RoSC.

### 2.2. Induction of Asphyxial CA

Asphyxial CA induction was performed according to published protocols [14,15,16]. In short, rats were anesthetized with 2.5% isoflurane in oxygen (33%) and nitrous oxide (67%), and the rats were endotracheally intubated with a 14-gauge cannula and ventilated to maintain respiration using a rodent ventilator (Harvard Apparatus, Holliston, MA, USA). An oxygen saturation probe of pulse oximetry (Nonin Medical Inc, Plymouth, MN, USA) was attached to the left foot to monitor peripheral oxygen saturation (SpO_2_). Body (rectal) temperature (37 ± 0.5 °C) was controlled during and after asphyxial CA surgery with a heat blanket. Electrocardiographic probes (GE healthcare, Milwaukee, WI, USA) were placed on the limbs for electrocardiogram (ECG), and the data were monitored during the CA. The left femoral artery was cannulated to monitor mean arterial pressure (MAP) (MLT 1050/D, AD Instruments, Bella Vista, Austria). The right femoral vein was cannulated to inject 2 mg of vecuronium bromide (GensiaSicor Pharmaceuticals, Irvine, CA, USA) intravenously 5 min after stabilization. The anesthesia and mechanical ventilation was stopped, and the endotracheal tube was disconnected from the ventilator. CA was defined when MAP was below 25 mmHg and subsequent pulseless electric activity was shown: CA was confirmed at 3–4 min after vecuronium bromide injection.

### 2.3. Cardiopulmonary Resuscitation (CPR)

CPR was performed according to published protocols [14,15,16]. In short, CPR was performed from 5 min after asphyxial CA as follows. The ventilator was reconnected, and 1 meq/kg of sodium bicarbonate (Daewon Pham, Seoul, Korea) and 0.005 mg/kg of epinephrine (Dai Han Pharm, Seoul, Korea) were injected and followed by mechanical ventilation with 100% oxygen. Mechanical chest compression was given at a rate of 300/min until MAP reached 60 mmHg and ECG activity was observed with palpable femoral artery pulse until RoSC. The animals requiring more than 5 min of CPR to achieve RoSC were excluded from this experiment: we used animals with 7–8 min of CA. When the animals were hemodynamically stable and breathed spontaneously around 1 h after RoSC, the catheters were removed, and the animals were extubated. Thereafter, the animals were kept in a thermal incubator (Mirae Medical Industry, Seoul, Korea) to maintain normal body temperature (37.0 ± 0.5 °C) or for hypothermic therapy. The rats of the sham group underwent the surgical procedure of asphyxial CA except the induction of CA.

### 2.4. Hypothermic Therapy

According to a published protocol [17], prompt hypothermia was induced after RoSC by surface cooling with isopropyl alcohol wipes. The target temperature was 33 ± 0.5 °C and was monitored by a rectal temperature sensor. Hypothermia was maintained for 4 h, and the animals were gradually re-warmed from 33 ± 0.5 °C to 37 ± 0.5 °C for 30 min using a warming blanket and hot pad. The 4-h duration [18] was chosen based on our pilot study showing a higher survival rate until 5 days after asphyxial CA compared with those of 1- or 2-h duration of hypothermia.

### 2.5. Tissue Preparation for Histology

Rats were anesthetized with sodium pentobarbital (60 mg/kg, i.p., JW Pharmaceutical, Seoul, South Korea) and transcardially perfused with 4% paraformaldehyde for the fixation of their brains and spinal cords. The brains and spinal cords were removed, embedded in tissue-freezing medium and serially sectioned into 30-μm coronal sections using a cryostat (Leica Microsystems GmbH, Wetzlar, Germany). For staining, sections were chosen at intervals of 120 μm in each animal.

### 2.6. Cresyl Violet (CV) and Fluoro-Jade B (F-JB) Histofluorescence Staining

CV (a marker for Nissl’s body) staining was carried out to observe cellular distribution or change in the brain and spinal cord as described in our published paper [14]. Briefly, the sections were stained with solution of 1.0% (*w*/*v*) CV acetate (Sigma–Aldrich, St. Louis, MO, USA).

F-JB (a high affinity fluorescent marker for the localization of neurodegeneration) histofluorescence staining was done to observe neuronal damage/death (degeneration) in the brain and spinal cord as described in our published paper [14]. In short, the sections were immersed in a solution of 0.0004% F-JB (Histochem, Jefferson, AR, USA) and placed on a slide warmer (50 ± 1 °C) to be reacted.

For the count of F-JB positive cells, F-JB positive cells were captured using an epifluorescent microscope (Carl Zeiss, Göttingen, Germany) with blue (450–490 nm) excitation light and a barrier filter, which was equipped with a digital camera (Axiocam, Carl Zeiss) connected to a PC monitor. The mean number of F-JB positive cells was obtained by averaging the total cell numbers obtained from each animal per group. All measurements were performed by two observers in blind conditions in order to ensure objectivity.

### 2.7. Immunohistochemistry for NeuN and Iba-1

To examine neuronal distribution and microgliosis following neuronal damage/death, immunohistochemistry for NeuN (a marker for neurons) and Iba-1 (a marker for microglia) were carried out, respectively, according to our published procedure [19]. In brief, the sections were incubated with primary mouse anti-NeuN (diluted 1:800, Abcam, Cambridge, MA, USA) or rabbit anti-Iba-1 (diluted 1:800, Wako, Osaka, Japan), reacted with secondary antibody (Vector Laboratories Inc., Burlingame, CA, USA) and developed by Vectastain ABC (Vector Laboratories Inc.). Finally, they were visualized with a solution of 3,3′-diaminobenzidine.

For the quantification of Iba-1 immunoreactivity, the images of the sections were captured using an AxioM1 light microscope (Carl Zeiss, Göttingen, Germany) equipped with a digital camera (Axiocam, Carl Zeiss) connected to a PC monitor. The images that were taken were calibrated into an array of 512 × 512 pixels under ×10 primary magnification. The immunoreactivity was measured by a 0–255 gray scale system and evaluated by optical density (OD), which was obtained after transformation of the mean gray level using a formula, OD = log (255/mean gray level). The ratio of the OD was calibrated as % (relative OD, ROD) using Adobe Photoshop (version 8.0) and analyzed using Image J 1.46 software (National Institutes of Health, Bethesda, MD, USA). The ratio of the ROD was calibrated as % compared with the control group designated as 100%. All measurements were performed by two observers in blind conditions in order to ensure objectivity.

### 2.8. Statistical Analysis

Data are expressed as the means ± standard error of the mean (SEM). Differences of the mean number or immunoreactivity of immunoreactive structures among the groups were statistically analyzed with analysis of variance followed by post hoc Bonferroni’s multiple comparison test using SPSS 18.0 (SPSS, Chicago, IL, USA). *p* < 0.05 was considered to indicate a statistically significant difference.

## 3. Results

### 3.1. Survival Rate

Survival rate in the CA groups was gradually reduced with time after CA, showing 66.7% at 12 h, 44.4% at 1 day, 18.2% at 2 days and 0% at 5 days after RoSC (Table 1). Survival rate in the CA/hypothermia group was significantly increased (100% at 12 h, 55.6% at 1 day, and 44.4% at 2 days and 33.3% at 5 days after RoSC) compared to those in the CA group; however, survival rate at 12 days after CA was 0% (Table 1).

### 3.2. Physiological Variables

Physiological variables in all animals were recorded in body weight, temperature, heart rate and MAP before the CA operation, as shown in Table 2. Physiological parameters in the sham, CA and CA/hypothermia groups before CA were similar to baselines. There were no significant differences in physiological variables between the survived animals of CA and CA/hypothermia groups at 12 h, 1 day and 2 days after CA (Table 2). Dead animals in each group showed unmeasurable data in heart rate and MAP before death.

### 3.3. CV Positive Cells

#### 3.3.1. Brain

**Figure 1:**
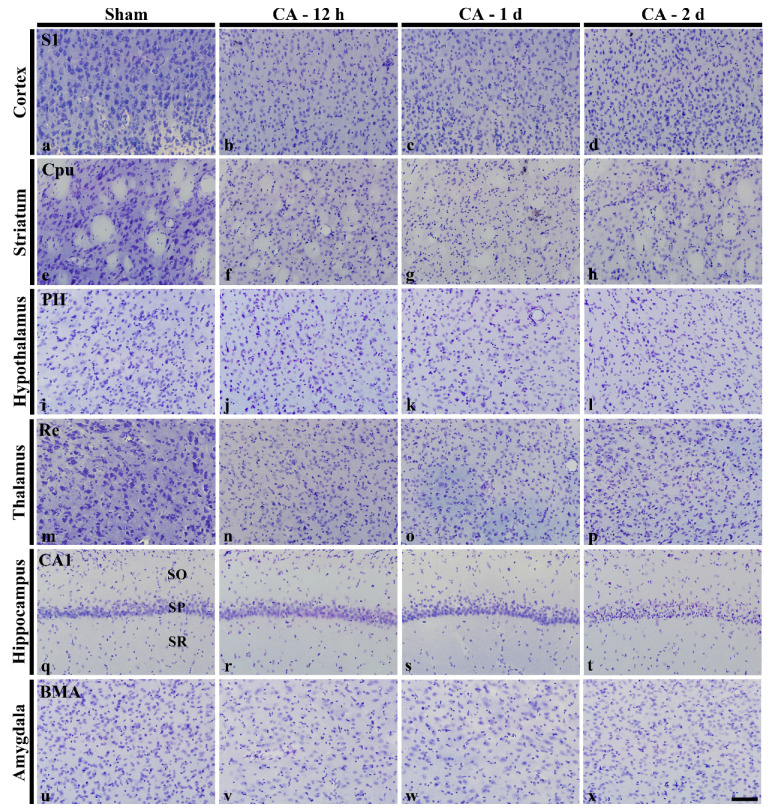
Cresyl Violet (CV) staining in main regions of the rat brain of the sham operated (**a**,**e**,**i**,**m**,**q**,**u**), and CA operated (**b**–**d**, **f**–**h**, **j**–**l**, **n**–**p**, **r**–**t**, **v**–**x**) groups. CV stainability is gradually pale (damaged) with time in the CA groups. Note that damage is distinct at 2 days after CA. BMA, basomedial amygdaloid nucleus; CA1, cornu ammonis 1; Cpu, caudate putamen; PH, posterior hypothalamic area; Re, reuniens thalamic nucleus; S1, primary somatosensory cortex; SO, stratum oriens; SP, stratum pyramidale; SR, stratum radiatum. Scale bar: 100 µm.

**Figure 2:**
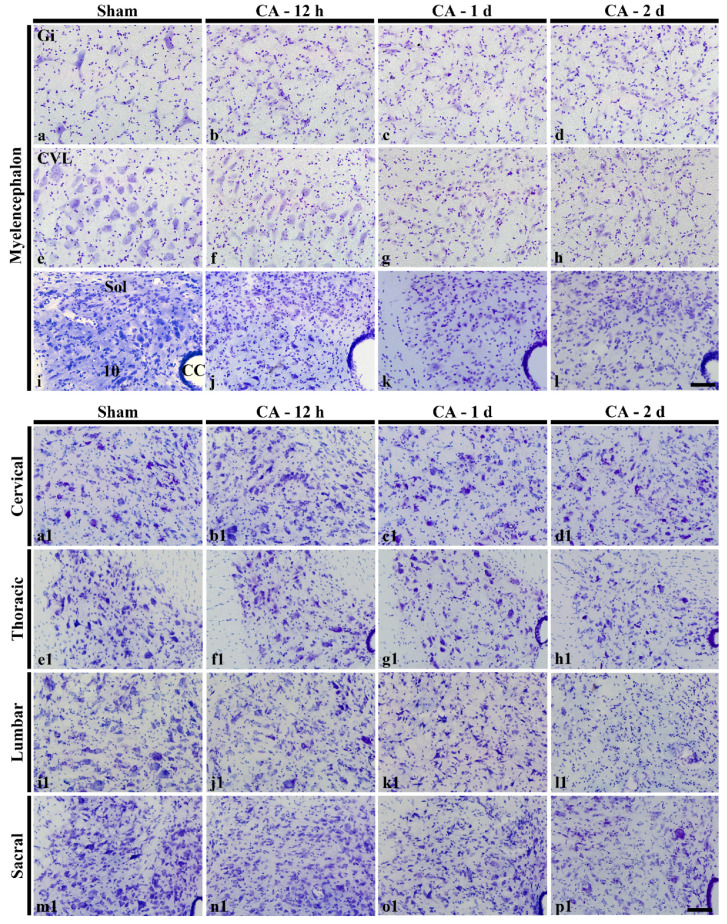
CV staining in the myelencephalon (**a**–**l**) and intermediate zone of the spinal cord (**a1**–**p1**) of the sham operated (**a**,**e**,**i**,**a1**,**e1**,**i1**,**m1**), and CA operated (**b**–**d**, **f**–**h**, **j**–**l**, **b1**–**d1**, **f1**–**h1**, **j1**–**l1**, **n1**–**p1**) groups. In the CA operated groups, CV positive cells are severely damaged in the myelencephalon and intermediate zone of the spinal cord at 2 days post-CA. 10, dorsal motor nucleus of vagus; CC, central canal; CVL, caudoventrolateral reticular nucleus; Gi, gigantocellular reticular nucleus; Sol, nucleus of solitary tract; Scale bars: 100 µm.

In the sham operated group, neurons in the brain showed normal characteristics with well-stained Nissl bodies in neuronal cytoplasm of the cortex (Figure 1a), striatum (Figure 1e), hypothalamus (Figure 1i), thalamus (Figure 1m), hippocampus (Figure 1q), amygdala (Figure 1u), and myelencephalon (Figure 2a,e,i).

In the CA operated groups, damage of CV positive cells was gradually enhanced (paler cytoplasm) with time after CA due to loss of Nissl substance in the cortex (Figure 1b–d), striatum (Figure 1f–h), thalamus (Figure 1n–p), hippocampus (Figure 1h–t), and myelencephalon (gigantocellular reticular nucleus in Figure 2b–d; caudoventrolateral reticular nucleus in Figure 2f–h; solitary nucleus and dorsal motor nucleus of vagus in Figure 2j–l).

#### 3.3.2. Spinal Cord

CV positive cells were scattered in the cervical, thoracic, lumbar, and sacral spinal cord of the sham operated groups (Figure 2a1,e1,i1,m1). In this study, among subregions (dorsal horn, intermediate zone and ventral horn) of the spinal gray matter, we examined neuronal damage in the intermediate zone containing neurons related with autonomic function. Like changes in the brain, the cytoplasm of CV positive cells in the intermediate zone was gradually damaged with time after CA (Figure 2b1–d1,f1–h1,j1–l1,n1–p1). In particular, CV positive cells were severely damaged in the intermediate zone of the thoracic, lumbar spinal and sacral spinal cord at 2 days after CA (Figure 2h1,l1,p1).

### 3.4. NeuN Positive Neurons and F-JB Positive Cells

**Figure 3:**
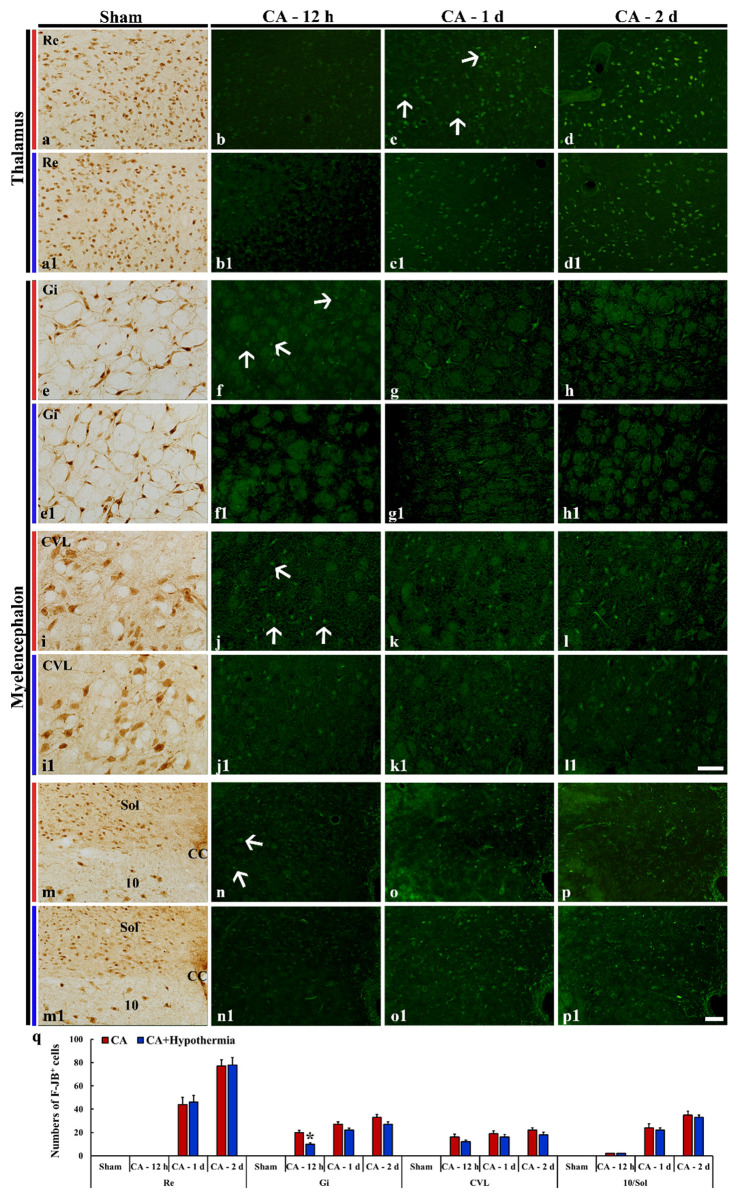
NeuN immunohistochemical staining in the thalamus (**a**,**a1**) and myelencephalon (**e**,**e1**,**i**,**i1**,**m**,**m1**) in the sham group, and F-J B histofluorescence staining in the thalamus (**b**–**d**, **b1**–**d1**) and myelencephalon (**f**–**h**, **f1**–**h1**, **j**–**l**, **j1**–**l1**, **n**–**p**, **n1**–**p1**) of the CA operated (**b**–**d**, **f**–**h**, **j**–**l**, **n**–**p**) and CA/hypothermia operated (**b1**–**d1**, **f1**–**h1**, **j1**–**l1**, **n1**–**p1**) groups. In the thalamus, F-JB positive cells (arrows) are shown and in the reuniens thalamic nucleus (Re) from 1 day post-CA. In the myelencephalon, F-JB positive cells (arrows) are found in the gigantocellular reticular nucleus (Gi), caudoventrolateral reticular nucleus (CVL), solitary nucleus (Sol) and dorsal motor nucleus of vagus (10). From 12 h post-CA, hypothermia significantly reduces the number of F-JB positive cells only in the Gi at 12 h post-CA. Scale bar = 100 µm. (**q**) mean numbers of F-JB positive cells in the CA and CA/hypothermia groups (*n* = 9 per group, * *p* < 0.05, vs. each CA group). The bars indicate the means ± SEM.

**Figure 4:**
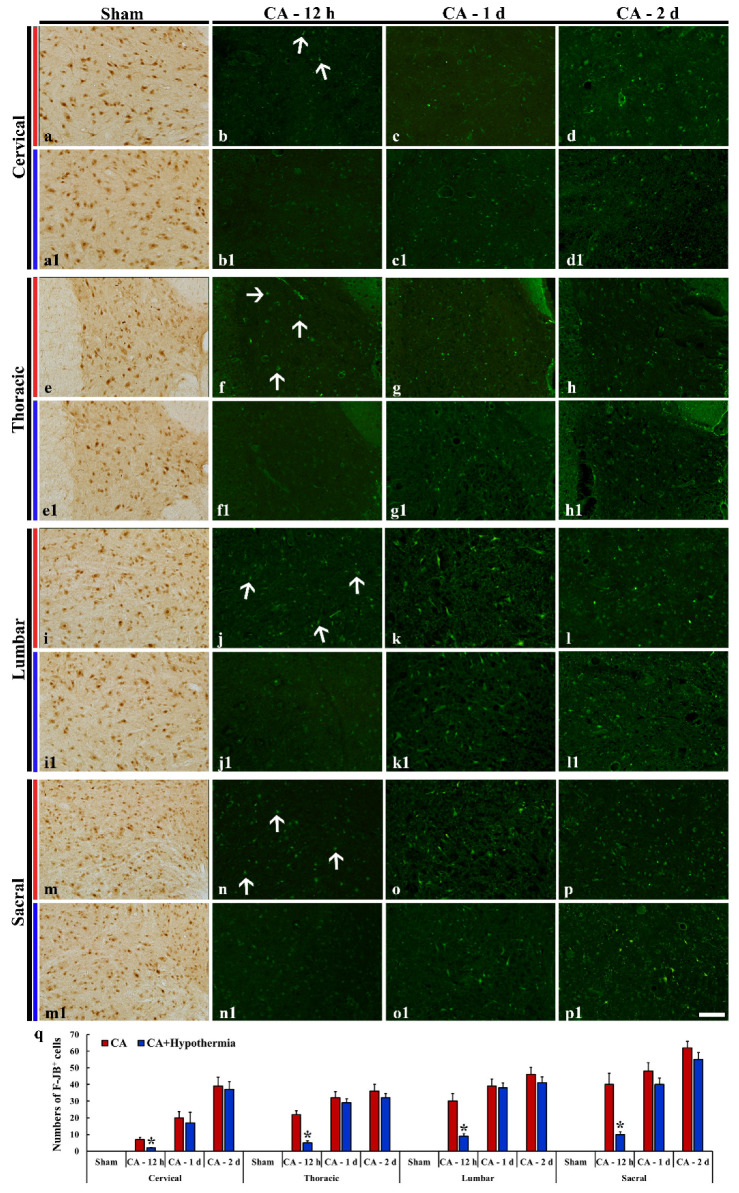
NeuN immunohistochemical staining in the intermediate zone of the spinal cord of the sham operated (**a**,**a1**,**e**,**e1**,**i**,**i1**,**m**,**m1**) and F-J B histofluorescence staining in the intermediate zone of the spinal cord of the CA operated (**b**–**d**, **f**–**h**, **j**–**l**, **n**–**p**), and CA/hypothermia operated (**b1**–**d1**, **f1**–**h1**, **j1**–**l1**, **n1**–**p1**) groups. Detection of F-JB positive cells (arrows) starts from 12 h post-CA, showing that numbers of F-JB positive cells are different between spinal cord levels. Note that hypothermia significantly reduces numbers of F-JB positive cells in all the levels only at 12 h after CA. Scale bars = 100 µm. (**q**) mean numbers of F-JB positive cells in the CA and CA/hypothermia groups (*n* = 9 per group, * *p* < 0.05, vs. each CA group). The bars indicate the means ± SEM.

Based on these findings of CV staining, we observed neuronal death (degeneration) in distinctively damaged areas (thalamus and myelencephalon in the brain and spinal cord) by using F-JB fluorescence staining (Figure 3 and Figure 4). In the results of F-JB fluorescence staining, degenerated neurons appear as a green fluorescent color, but if there are no degenerated neurons, the result shows a black background. Therefore, in this study, we showed the distribution of neurons stained with NeuN (a marker for neuronal nuclei) in the sham group (Figure 3a,a1,e,e1,i, i1,m,m1 and Figure 4a, a1,e,e1,i,i1,m,m1) and neurons stained with F-J B (a marker for degenerated neurons) in the ischemia group.

#### 3.4.1. Brain

No F-JB positive cells were found in any regions in the brain of the sham operated group (data not shown). At 12 h after CA, F-JB positive cells started to be found in the myelencephalon: several F-JB positive cells in the gigantocellular reticular nucleus (Figure 3f) and caudoventrolateral reticular nucleus (Figure 3j), and a few F-JB positive cells in the solitary nucleus and dorsal motor nucleus of vagus (Figure 3n). At 1 day after CA, many F-JB positive cells started to be shown in the reuniens thalamic nucleus (Figure 3c). In addition, at this point in time, numbers of F-JB positive cells were more increased in the gigantocellular reticular nucleus (Figure 3g), caudoventrolateral reticular nucleus (Figure 3k), solitary nucleus and dorsal motor nucleus of vagus (Figure 3o) compared with those at 12 h post-CA (Figure 3q). At 2 days after CA, the number of F-JB positive cells were further increased in all of the nuclei (Figure 3d,h,l,p) compared to those at 1 day post-CA (Figure 3q).

In the CA/hypothermia groups, numbers of F-JB positive cells were not altered or deceased according to the nuclei compared to those in the CA groups. In the reuniens thalamic nucleus, numbers of F-JB positive cells were not different from those of the CA groups at all points in time after CA (Figure 3c1,d1,q). In the gigantocellular reticular nucleus, numbers of F-JB positive cells were significantly decreased at 12 h post-CA, but not at 1 and 2 days post-CA (Figure 3f1,g1,h1,q). In the caudoventrolateral reticular nucleus, numbers of F-JB positive cells were not significantly decreased at all points in time after CA (Figure 3j1–l1,q). Also, in the solitary nucleus and dorsal motor nucleus of vagus, numbers of F-JB positive cells were not significantly decreased at all points in time after CA (Figure 3n1–p1,q).

#### 3.4.2. Spinal Cord

In the sham operated group, F-JB positive cells were not found in the intermediate zone as well as the other subregions of the cervical, thoracic, lumbar and sacral spinal cord (data not shown). At 12 h after CA, a few F-JB positive cells were shown in the intermediate zone of the cervical spinal cord (Figure 4b), whereas, many F-JB positive cells were detected in the intermediate zone of the thoracic (Figure 4f), lumbar (Figure 4j), sacral (Figure 4n) and spinal cord (Figure 4q). Thereafter, numbers of F-JB positive cells in the intermediate zone were gradually increased in all the spinal levels (Figure 4c,d,g,h,k,l,o–q), showing that the number of F-JB positive cells was highest in the sacral level at 2 days post-CA (Figure 4q).

At 12 h after hypothermic therapy, numbers of F-JB positive cells were significantly reduced in the intermediate zone of all the spinal levels (Figure 4b1,f1,j1,n1,q). Thereafter, the number of F-JB positive cells in each spinal level was lower than that CA operated group, but the reduced number was not significant compared with that in the untreated group (Figure 4c1,d1,g1,h1,k1,l1,o1,p1,q).

### 3.5. Iba-1 Immunoreactivity

Based on findings of F-JB fluorescence staining, we examined microgliosis in the distinctively damaged areas (thalamus, myelencephalon and spinal cord) by Iba-1 immunohistochemistry.

#### 3.5.1. Brain

**Figure 5:**
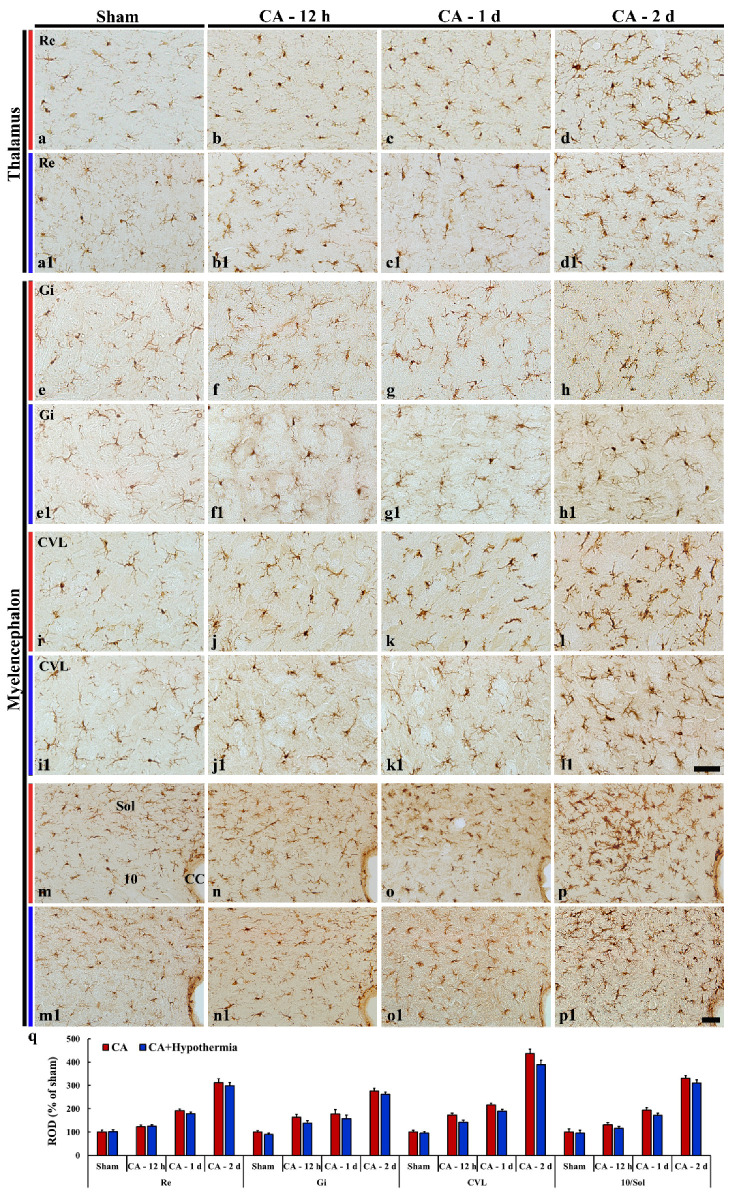
Iba-1 immunohistochemical staining in the thalamus (**a**–**d**) and myelencephalon (**e**–**p**) of the sham operated (**a**,**a1**,**e**,**e1**,**i**,**i1**,**m**,**m1**), CA operated (**b**–**d**, **f**–**h**, **j**–**l**, **n**–**p**), and CA/hypothermia operated (**b1**–**d1**, **f1**–**h1**, **j1**–**l1**, **n1**–**p1**) groups. Typical resting form of Iba-1 immunoreactive microglia are found in the sham operated group. After CA, Iba-1 immunoreactive microglia are morphologically activated, and Iba-1 immunoreactivity is increased with time after CA. Iba-1 immunoreactivity in the CA/hypothermia operated group is not significantly different comparted to that in the CA operated group. 10, dorsal motor nucleus of vagus; CVL, caudoventrolateral reticular nucleus; Gi, gigantocellular reticular nucleus; Re, reuniens thalamic nucleus; Sol, nucleus of solitary tract. Scale bars = 50 µm. (**q**) Quantitative analysis of Iba-1 immunoreactivity in the CA and CA/hypothermia groups (*n* = 9 per group). The bars indicate the means ± SEM.

In the sham operated group, Iba-1 immunoreactive microglia were shown throughout the thalamus and myelencephalon, showing that they displayed small round cell body with thin ramified processes (Figure 5a,e,i,m). Twelve hours after CA, Iba-1 immunoreactive microglia showed enlarged cytoplasm with thick processes, and the Iba-1 immunoreactivity was increased compared to those in the sham operated group in the myelencephalon (Figure 5f,j,n,q), but not in the thalamus (reuniens thalamic nucleus) (Figure 5b,q). At 1 and 2 days after CA, immunoreactive microglia were morphologically activated and showed hypertrophied cytoplasm with short and thickened processes in the thalamus (Figure 5c,d), and myelencephalon (Figure 5g,h,k,l,o,p). In particular, Iba-1 immunoreactivity was significantly increased in the reuniens thalamic nucleus, gigantocellular reticular nucleus, caudoventrolateral reticular nucleus, solitary nucleus and dorsal motor nucleus of vagus at 2 days after CA (Figure 5q).

In the CA/hypothermia group, Iba-1 immunoreactive microglia were similar to those in the CA group: namely, the Iba-1 immunoreactivity was increased in a time-dependent manner in the reuniens thalamic nucleus (Figure 5b1–5d1), gigantocellular reticular nucleus (Figure 5f1–5h1), caudoventrolateral reticular nucleus (Figure 5j1–5l1), solitary nucleus and dorsal motor nucleus of vagus (Figure 5n1–5p1). When the ROD in the CA/hypothermia group was compared with that in the CA group, significant difference was not found between the two groups (Figure 5q).

#### 3.5.2. Spinal Cord

**Figure 6:**
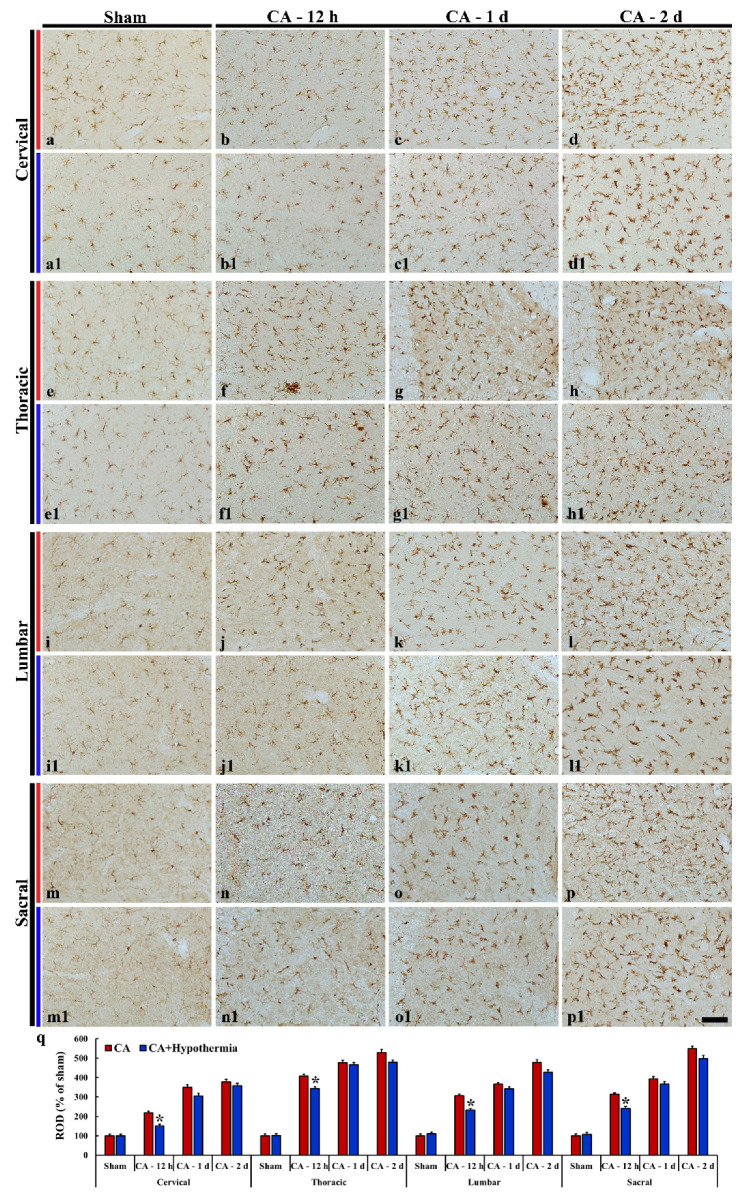
Iba-1 immunohistochemical staining in the spinal cord of the sham operated (**a**,**a1**,**e**,**e1**,**i**,**i1**,**m**,**m1**), CA operated (**b**–**d**, **f**–**h**, **j**–**l**, **n**–**p**), and CA/hypothermia operated (**b1**–**d1**, **f1**–**h1**, **j1**–**l1**, **n1**–**p1**) groups. After CA, Iba-1 immunoreactivity is increased with time. Hypothermia significantly decreases microgliosis in all spinal levels only at 12 h after CA. Scale bars = 50 µm. (**q**) Quantitative analysis of Iba-1 immunoreactivity in the spinal cord in the CA and CA/hypothermia groups (*n* = 9 per group, * *p* < 0.05, vs. each CA group). The bars indicate the means ± SEM.

Iba-1 immunoreactive microglia in the sham operated group scattered in the intermediate zone in all (cervical, thoracic, lumbar and sacral) spinal levels, showing that they had small cytoplasm with long branched processes (Figure 6a,e,i,m). In the CA groups, Iba-1 immunoreactive microglia became hypertrophied in the cell bodies and shortened and swollen in the processes at 12 h after CA (Figure 6b,f,j,n), showing that the ROD was significantly increased (Figure 6q). Thereafter, Iba-1 immunoreactive microglia were more activated in all of the levels with time after CA (Figure 6c,g,k,o,q).

In the CA/hypothermia group, the activation of Iba-1 immunoreactive microglia was significantly attenuated in all of the levels at 12 h after CA compared with that in the CA group (Figure 6b1,f1,j1,n1,q). However, in all of the levels at 1 and 2 days after CA, the Iba-1 immunoreactivity (ROD) of the CA/hypothermia group was not different from that in the CA group (Figure 6c1,d1,g1,h1,k1,l1,o1,p,q).

### 3.6. Drawings of Time-Course Damage in the CNS after CA

**Figure 7:**
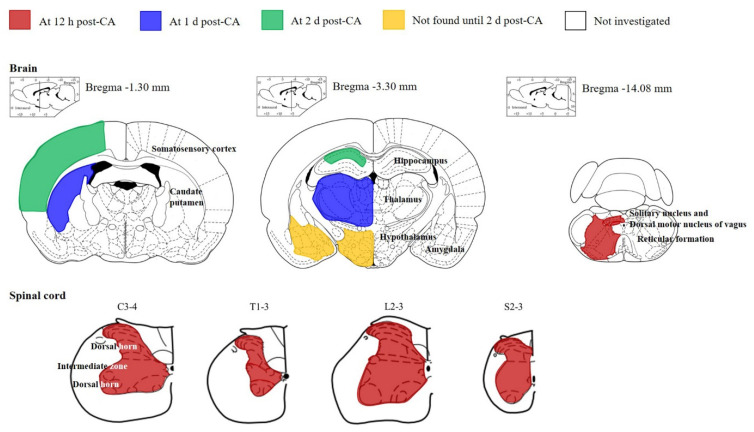
Schematic drawings of neuronal damage/death in brain and spinal cord regions related to autonomic control in the rat. Neuronal death at 12 h, 1 day and 2 days post-CA is marked by red, blue and green, respectively. Yellow indicates that neuronal death is not shown until 2 days post-CA, and white indicates that we did not investigate neuronal death after CA. Generally, the earliest neuronal death occurs in the myelencephalon and spinal cord.

We observed changes in neurons (neuronal damage/death) by CV staining and FJB immunofluorescence staining and microgliosis by Iba-1 immunohistochemistry in the regions related to autonomic function in the CNS after CA. In the brain, the cingulate cortex, hippocampus, thalamus, hypothalamus, amygdala and myelencephalon were selected, and, in the spinal cord, cervical, thoracic, lumbar and sacral levels were selected (Figure 7). As shown in Figure 7, the earliest neuronal damage/death in the CNS after CA was found in the myelencephalon and spinal cord, which contain neurons related with autonomic lower motor neurons. These schematic drawings are of great service to everyone who is related to researches, therapy and prevention of CA.

## 4. Discussion

In the present study, we investigated the mortality rate of asphyxial CA-induced rats and examined neuronal damage and death and microgliosis in the brain and spinal cord using F-JB staining and Iba-1 immunoreactivity after CA. In addition, to examine which regions in the CNS correlate with death following CA, we investigated the effects of hypothermia on the mortality rate, neuronal damage/death and microgliosis after asphyxial CA.

In this study, the survival rate of the rats with CA dropped to less than 50% at 1 day, was about 18% at 2 days, and 0% at 5 days after asphyxial CA for 7–8 min. Jia et al. (2006) reported a mean survival of 66.9 h (around 2.7 days) after 7 min of asphyxial CA in Wistar rats [20], and halothane (4.5%, O_2_/N_2_O 50/50%) was used as an anesthetic agent and vecuronium (2 mg/kg) was also used in the rats. However, in previous studies reporting a high survival rate after CA, Handrickx et al. (1984) used halothane (O_2_/N_2_O 50/50%) as an anesthetic and pancuronium (0.4 mg) for muscle relaxation in a Sprague-Dawley rat model of asphyxial CA for 6 min [6]. Cohan et al. (2015) used 4% isoflurane (O_2_/N_2_O 30/70%) as an anesthetic and vecuronium bromide (2.0 mg/kg) in a Fischer 344 rat model of asphyxial CA for 6 min [21]. In addition, Keilhoff et al. (2020) used 5% sevoflurane (O_2_/N_2_O 40/60%) as an anesthetic and vecuronium (1 mg/kg) in a Wistar rat model of asphyxial CA for 6 min [22]. The difference in mortality rates may be due to the different experimental methods, such as the duration of CA, the types of animal model of CA, body temperature after CA, and the types of anesthetic used. However, the precise reason for this discrepancy is beyond the scope of our knowledge. This is a limitation of our current research. In our opinion, the important factors in the mortality rate after CA must depend up on the duration of CA and body temperature after CA. In addition, there are many methods to establish experimental models of CA, but most of the studies were conducted with approximately 10 animal samples, so studies with larger sample sizes could produce more accurate results.

To investigate the factors affecting of the survival rate after CA, we examined the vulnerable areas in various brain regions related to autonomic function including the cingulate cortex, hippocampus, thalamus, hypothalamus, amygdala, myelencephalon, and the spinal cord after CA using F-JB fluorescence staining. We found that F-JB positive cells (dead cells) were found in several nuclei (the gigantocellular reticular nucleus, caudoventrolateral reticular nucleus, solitary nucleus, and dorsal motor nucleus of vagus) located in the myelencephalon, and the intermediate zone in all of the spinal cord levels (the cervical, thoracic, lumbar, and sacral levels) at the earliest time (12 h) after CA. In addition, the number of F-JB positive cells was significantly increased in those nuclei or areas with time after CA. In previous studies, ischemic brain damage after whole body ischemia due to asphyxial CA in rats was reported in the thalamic reticular nucleus 6 h post-CA [23], in the intermediate gray matter of the lumbar spinal cord at 12 h post-CA [24], and in the hippocampal CA1 region 1 day post-CA [23,24] using the TUNEL method. In addition, Katz et al. (1995) reported that neuronal damage after asphyxial CA in rats was found in the cortex, caudate putamen, thalamus, and cerebellum at 3 days post-CA [25]. Furthermore, Lin et al. (2013) reported that asphyxial CA in rats caused neuronal changes (shrunken eosinophilic neurons) in the cortex and hippocampus 7 days post-CA [1]. Based on the findings of the previous and our current studies, it is likely that asphyxial CA produces neuronal damage in many CNS regions including the cerebral cortex, thalamus, hippocampus, caudate putamen, cerebellum, and spinal cord within 7 days after asphyxial CA. In addition, our current results showed that neuronal death (loss) in several nuclei of the myelencephalon (the gigantocellular reticular nucleus, caudoventrolateral reticular nucleus, solitary nucleus and dorsal motor nucleus of vagus) and in the lamina VII (intermediate zone) of all spinal levels significantly preceded that in the cortex, hippocampus, thalamus, hypothalamus, and amygdala following asphyxial CA. This finding means that the above-mentioned nuclei or areas are the most vulnerable to CA. In contrast, the areas related to autonomic function, including the hippocampus, amygdala, thalamus, and hippocampus, showed later or no neuronal death (loss) after CA. To the best of our knowledge, this was the first study to show that neuronal death (loss) following asphyxial CA in rats happened earliest in several nuclei in the myelencephalon and in the intermediate zone of all spinal cord levels.

The novel aspect of our current findings is that, as described above, the earliest neuronal loss happened in the nuclei of the myelencephalon and spinal lamina VII after CA. These neurons are autonomic lower motor neurons and life depends upon these neurons. Medullary reticulospinal tracts, which are located in the myelencephalon and involved in automatic breathing, arise from the medullary reticular formation (gigantocellular nucleus) and terminates in the spinal lamina VII [26]. In addition, bulbospinal tracts play important roles in autonomic function, motor control, and sensory input (including pain) via coordinating the activity of spinal networks through the caudoventrolateral reticular nucleus [27]. Namely, the ventral respiratory columns located in the myelencephalon generate rhythmic motor patterns of inspiratory and expiratory activity during normal breathing [9], and the caudoventrolateral reticular nucleus, a compartment of the ventral respiratory columns, contains bulbospinal expiratory neurons that project to thoracic, lumbar, and phrenic expiratory motor neurons (premotor interneurons) located within the spinal intermediate gray matter (lamina VII) [9,27]. It has been reported that dysfunction in the respiratory and cardiovascular systems during the post-resuscitation phase after CA is highly correlated with low survival rate [1,8].

The homeostasis of cardiac physiology is mainly controlled by the autonomic nervous system located in the hypothalamus, myelencephalon and spinal cord [28]. To be specific, the cardiac sympathetic nervous system, which originates from neurons in the paraventricular nucleus of the hypothalamus and the intermediate zone of the upper thoracic spinal cord (T1–T4), increases heart rate and cardiac contractility, whereas the cardiac parasympathetic nervous system, which emerges from the dorsal vagal motor nucleus of the vagus nerve in the myelencephalon, suppresses heart rate and blood pressure [28,29]. The hypothalamic paraventricular nucleus is not only an important integrative site regulating autonomic and cardiovascular activities but is also one of the main components of the central neurocircuitry of cardiac sympathetic afferent reflexes [30]. In our current study, we did not find F-JB positive cells (dead neurons) in the paraventricular nucleus of the hypothalamus until 2 days after CA, whereas extensive F-JB positive cells were found in the spinal lamina VII as well as in several nuclei of the myelencephalon 12 h after CA. It has been reported that neurons located in the hypothalamus were more resistant to oxygen deprivation than those located in the cerebral cortex or hippocampus [31]. Brisson et al. (2013) reported that the hypothalamic neurons following ischemia exhibited higher resistance to ischemia than that of thalamic neurons due to relatively good Na^+^/K pump function during ischemia [32]. Taken together, our present results suggest that the earliest neuronal degeneration in the respiratory system including the bulbospinal tracts (between the gigantocellular reticular nucleus and the caudoventrolateral reticular nucleus of the myelencephalon and the spinal lamina VII) and cardiovascular system (between the vagal motor nucleus of the myelencephalon and the spinal lamina VII) due to asphyxial CA may contribute significantly to the high mortality rate after CA in rats.

The activation of microglia, reflecting the inflammatory response in the central nervous system, is one of the key processes correlated with ischemic neuronal death because they release cytotoxic and pro-inflammatory cytokines such as interleukin (IL)-1β, tumor necrosis factor (TNF)-α, and reactive oxygen species [33,34,35]. CA was reported to increase activated Iba-1 immunoreactive microglia [2,36], and microglial TNF-α and IL-1β gene expression levels [37] in the rat brain. Consistent with these established studies, in our current study, Iba-1 immunoreactivity was markedly increased in relation to neuronal death in the above-mentioned nuclei or areas and was highest 2 days post-CA. It is well known that ischemic injury reliably leads to microglial activation and proliferation along with neuronal death [19,38]. Thus, many studies have suggested that blocking microglia activation promotes neuroprotection and may be used to treat ischemic brain injury [39,40]. In this sense, hypothermic therapy as post-CA care is one of the positive contributing factors improving survival after CA [17,41], which is closely linked to decreases in inflammatory markers (i.e., attenuation of the activity of microglia) [42,43] or inhibition of inflammasome component expression [44]. However, some previous studies demonstrated that hypothermia reduced microglial activation, but not ischemic neuronal death in the hippocampus 3 or 7 days after CA [45,46]. In our current study, we found that hypothermic therapy significantly reduced microglial activation in the spinal intermediate zone only 12 h after CA, but not in the myelencephalon, and thereafter, microglia activation was not attenuated in the spinal cord. With this finding, neuronal death in the spinal intermediate zone was temporarily attenuated only 12 h after CA, and thereafter, neuronal death in the intermediate zone was not different from the CA group. Furthermore, the survival rate in the CA/hypothermia group was temporarily increased until 5 days after CA, but the survival rate at 12 days after CA was 0%. These results suggest that the increased survival rate until 5 days in the hypothermia/CA group was closely associated with temporarily reduced ischemic damage in the spinal cord and myelencephalon at early points after CA.

In summary, the findings in this study showed that the earliest neuronal death occurred in the gigantocellular reticular nucleus, caudoventrolateral reticular nucleus, solitary nucleus, and dorsal motor nucleus of vagus located in the myelencephalon, and in the intermediate zone (lamina VII) of all spinal levels after CA in rats. These neurons, observed to be autonomic lower motor neurons, are associated with respiration. Therefore, their earliest loss or death following CA may be one of the major causes of the high mortality rate after CA. Taken together, this study indicated that hypothermic therapy temporarily increased the survival rate by attenuating or delaying neuronal death in the spinal cord and myelencephalon at early times but not later.

## Figures and Tables

**Table 1:** Survival (%) rate in the sham, cardiac arrest (CA) and CA/hypothermia groups.

	Post-CA 12 h (*n* = 9)	Post-CA 1 d (*n* = 9)	Post-CA 2 d (*n* = 9)	Post-CA 5 d	Post-CA 12 d
Sham	100 (*n* = 5/5)	100 (*n* = 5/5)	100 (*n* = 5/5)	100 (*n* = 5/5)	100 (*n* = 5/5)
CA	66.7 (*n* = 6/9)	44.4 (*n* = 4/9)	18.2 (*n* = 2/11)	0 (*n* = 0/9)	-
CA/hyperthermia	100 (*n* = 9/9)	55.6 (*n* = 5/9)	44.4 (*n* = 4/9)	33.3 (*n* = 3/9)	0 (*n* = 0/9)

**Table 2:** Physiological variables in the sham, CA and CA/hypothermia groups.

**CA**	**Sham** **(*n* = 5)**	**Post-CA 12 h** **(*n* = 6)**	**Post-CA 1 d** **(*n* = 4)**	**Post-CA 2 d** **(*n* = 2)**	**Post-CA 5 d** **(*n* = 0)**
Body weight (g)	309.8 ± 6.1	289.8 ± 7.9	304.9 ± 6.9	308.6 ± 6.8	-
Temperature (°C)	36.9 ± 0.1	36.4 ± 0.3	36.2 ± 0.1	36.1 ± 0.2	-
Heart rate (beats/min)	336 ± 15	358 ± 36	354 ± 46	347 ± 32	-
MAP (mmHg)	114 ± 17	116 ± 26	118 ± 13	120 ± 18	-
**CA/hypothermia**	**Sham** **(*n* = 5)**	**Post-CA 12 h** **(*n* = 9)**	**Post-CA 1 d** **(*n* = 5)**	**Post-CA 2 d** **(*n* = 4)**	**Post-CA 5 d** **(*n* = 3)**
Body weight (g)	298.2 ± 11.6	300.4 ± 17.4	303.8 ±16.8	302 ± 15.6	300 ± 10.2
Temperature (°C)	36.9 ± 0.1	36.4 ± 0.3	36.2 ± 0.1	36.1 ± 0.2	36.2 ± 0.4
Heart rate (beats/min)	345 ± 15	347 ± 42	352 ± 38	343 ± 35	345 ± 27
MAP (mmHg)	116 ± 11	108 ± 13	115 ± 12	118 ± 10	114 ± 11

Data are expressed as the mean ± SD for each group. CA, asphyxial cardiac arrest; MAP, mean arterial pressure.

## Data Availability

The data presented in this study are available in an insert article.
